# Biopsy Needle Integrated with Rotational Oblique Spectral Ultrasound (ROSUS) Imaging

**DOI:** 10.3390/s26041098

**Published:** 2026-02-08

**Authors:** Benjamin C. Kreager, Wei-Yi Chang, Jian Tian, Huaiyu Wu, Xiaoning Jiang

**Affiliations:** 1 Department of Mechanical and Aerospace Engineering, North Carolina State University, Raleigh, NC 27695, USA; bkreage@ncsu.edu; 2 Nordson Acoustic Microscopy Division, Elk Grove Village, IL 60007, USA; wei-yi.chang@nordsonsonoscan.com; 3 CTS Advanced Materials, Lisle, IL 60532, USA; jian.tian@ctscorp.com

**Keywords:** spectral ultrasound imaging, blood vessel detection, fine needle aspiration, high-frequency ultrasound, single-element ultrasound

## Abstract

Biopsy procedures are essential for definitive cancer diagnosis but remain limited by the risk of accidental blood vessel puncture, which can lead to hemorrhage and procedural failure. Conventional imaging guidance often provides insufficient vascular contrast, making vessel avoidance during needle insertion challenging. A rotational oblique spectral ultrasound (ROSUS) imaging system was developed to improve vessel detection and needle guidance during biopsy procedures. The device integrates a high-frequency PMN-PT 1–3 composite transducer mounted at a 45° angle within the 18-gauge needle tip, enabling simultaneous forward- and side-looking capability. By combining synchronized rotational–axial scanning with multifrequency signal ratio (MFSR) processing, ROSUS achieved volumetric images with blood–tissue contrast ratio improvement over 1.2 dB compared to conventional B-mode signal processing while maintaining high spatial resolution of 85 µm and 424 µm in axial and lateral directions, respectively. These results demonstrate that frequency-domain spectral processing can improve vessel and tissue differentiation, offering an 18-gauge needle-integrated platform for safer and more accurate biopsy needle-based procedures.

## 1. Introduction

Cancer continues to be one of the leading global causes of mortality, significantly impacting public health. In 2022, worldwide cancer deaths reached nearly 10 million, while over 20 million new cases were reported [1]. Early detection and precise diagnosis are critical for improving patient outcomes, allowing timely intervention and targeted treatments to mitigate spread of the disease via metastasis [2,3,4,5,6]. Percutaneous needle biopsy procedures have become standard practice for diagnosing abnormalities within soft tissues, including the liver, kidneys, lungs, and lymph nodes [7,8,9,10,11,12]. These minimally invasive biopsies play an essential role in confirming malignancies and guiding clinical decision making, thus profoundly affecting patient prognosis and treatment planning [13,14]. Despite their extensive clinical use and diagnostic accuracy, needle biopsy procedures carry significant risks. A major concern associated with these interventions is the accidental puncture of blood vessels during needle insertion, potentially leading to severe complications such as internal bleeding, hematoma formation, and failed procedures [15,16,17,18,19,20,21]. Clinically significant post-procedural pseudoaneurysm or hemorrhage typically arises from injury to arterial branches with diameters of approximately 1–4 mm, such as intercostal or inferior epigastric vessels [15,21,22,23,24,25,26]. The inadvertent puncture of vessels often results in prolonged hospital stays, requirement of additional medical interventions, and a consequent delay in patient recovery [27,28]. Thus, reducing such complications is paramount for improving procedural safety and patient outcomes.

To enhance guidance of the biopsy needle to the target tissue, various medical imaging modalities are currently utilized. These include ultrasound (US), computed tomography (CT), fluoroscopy, and magnetic resonance imaging (MRI) [29,30,31,32]. CT guidance has become a well-established technique, but it exposes patients to ionizing radiation [33,34]. Additionally, CT guidance suffers from low contrast in soft tissues, resulting in poor vessel visualization [35]. Similar to CT guidance, fluoroscopy also exposes patients to ionizing radiation and has low contrast sensitivity between adjacent tissues without iodine-based contrast agents [36]. Closed-bore MRI restricts operator access and tool maneuverability, while open MRI improves access at the cost of image quality, which complicates trajectory modification to avoid blood vessels [37,38]. US is non-ionizing, portable, relatively low-cost, and has a high frame rate, allowing rapid needle trajectory changes. Therefore, external ultrasound imaging is considered the gold standard for needle biopsy guidance [39,40,41].

While external US is widely adopted for percutaneous needle biopsy guidance, it still has its shortcomings—especially pertaining to its blood vessel detection capabilities. Typical US arrays used for biopsy needle guidance have center frequencies less than 15 MHz, with lower frequencies (3–6 MHz) being used for deeper abdominal or thoracic targets while higher frequencies (7–15 MHz) are utilized for shallower targets, such as breast, thyroid, or lymph nodes [42,43]. These frequency ranges are strategically selected due to the trade-off between axial resolution and signal penetration depth with ultrasound transducers. Axial resolution improves with increases in center frequency due to its relationship with the acoustic wavelength [44]. Conversely, signal attenuation has an approximately linear relationship with frequency in soft tissues (~0.7 dB/cm MHz), which degrades signal-to-noise ratio (SNR) and, hence, obscures tissue structural features in deeper regions, limiting penetration depth [45,46]. Within the 15 MHz frequency regime used for external US-imaging-based needle biopsy guidance, red blood cells (RBCs) are much smaller than the wavelength, and exhibit low backscattering, which causes the lumens to appear with minimal contrast compared to surrounding tissue unless the vessel walls are well aligned with the transducer aperture [47]. To solve the problem induced by low contrast between the vessels and tissue, color/power Doppler or contrast-enhanced ultrasound (CEUS) is used [8,42,48]. Color Doppler is useful for identifying flow direction and blood vessels, but it is sensitive to the angle of the transducer with respect to the vessel, which may result in false negatives for unfavorable vessel orientations [49,50]. Power Doppler signals are less effected by the angle between the transducer and the vessel but suffer from sensitivity to motions such as respiration or needle movement within tissue [51,52,53]. CEUS adds contrast with intravascular microbubbles, making perfused vessels and viable tissue conspicuous when grayscale/Doppler are equivocal [54,55,56]. However, using CEUS adds further complication to the biopsy procedure by requiring bolus injection or intravenous infusion of microbubbles [54].

To mitigate existing limitations with external ultrasound guidance, several studies have leveraged minimally invasive devices to bring sensing or imaging modalities closer to the blood vessels. These include electrical bioimpedance sensing at the needle tip, intravascular ultrasound (IVUS), endobronchial ultrasound (EBUS) or endoscopic ultrasound (EUS) with Doppler, photoacoustic catheters/needles, and optical coherence tomography (OCT) needles [57,58,59,60,61,62,63,64,65]. Bioimpedance sensing at the needle tip accurately discriminates between various soft biological tissues such as blood, vessel walls, fat and muscle, but the measurement region is confined to the vicinity immediately surrounding the needle tip and it has limited spatial information [57,58]. IVUS, EBUS with Doppler, and EUS with Doppler are useful for intravenous diagnosis or vessel detection but are application-limited and would not be useful for all percutaneous biopsy procedures. Furthermore, Doppler imaging requires an ultrasound array, which is difficult to integrate with a needle due to high numbers of ultrasound elements and space constraints for wire paths within the needles [66,67]. Photoacoustic-imaging-based catheters and needles have been shown to provide sufficient contrast between the arterial lumen and surrounding tissues due to high absorption of light in hemoglobin found within blood [62,63]. Yet, photoacoustic imaging still has drawbacks—depth penetration is hindered by optical scattering and absorption in blood and tissue, and the integration of the laser, optical paths, ultrasound transducer, and their alignment requirement results in a complicated system arrangement [68,69]. A side-looking OCT needle has also been developed for blood vessel detection and discerns the vessel from surrounding tissue with high sensitivity and specificity (up to 91.2% and 97.7%, respectively). However, it does not have forward-viewing capabilities and would not allow operators to see and avoid vessels directly in the path of the needle [64].

To respond to the clinical needs mentioned above, minimally invasive imaging technology is required which has forward- and side-viewing capabilities and design simplicity, sufficiently distinguishes between blood vessels and surrounding tissue, and overcomes depth penetration limitations. Our previous work describes the integration of a side-looking single-element 50 MHz ultrasound transducer embedded in the tip of the biopsy needle for fine needle aspiration (FNA) procedures [67]. This design brought the high-frequency transducer closer to the target lesion, mitigating attenuation effects while maintaining high axial resolution for detecting potential micrometastases. Yet, our previous design had some drawbacks with regard to its side-looking transducer orientation and blood vessel detection capabilities [67,70,71,72,73,74,75,76,77,78,79,80,81]. The side-looking transducer configuration could not detect vessels in the path of needle insertion, and high-frequency transducers have innate difficulties detecting blood vessels with traditional B-mode imaging due to high backscattering in blood at high frequencies, which obscures the arterial lumen [70,82,83,84,85].

To overcome the limitations, rotational oblique spectral ultrasound (ROSUS) imaging is introduced, a needle-integrated minimally invasive approach that combines oblique transducer angle with spectral-domain signal processing to improve vessel detection and needle guidance. A high-frequency single-element transducer is mounted at a 45° angle in the tip of the needle, producing volumetric images when rotated and linearly advanced, capturing forward- and side-viewing perspectives. This hardware configuration is paired with multifrequency signal ratio (MFSR) processing, which enhances contrast between blood and tissue. These features collectively enable ROSUS to map vessels along the needle trajectory while maintaining high-resolution imaging of the target lesion during the biopsy procedure. Unlike Doppler, IVUS, OCT and photoacoustic approaches, ROSUS does not rely on flow detection, intravascular access, optical component assembly, or contrast agents. In this study, the system design is described, imaging performance is characterized in blood–tissue phantoms, and its feasibility to improve safety and tissue acquisition accuracy in needle biopsy procedures is demonstrated. The remainder of this paper describes the transducer design and ROSUS methodology, followed by experimental validation, discussion, and conclusions.

## 2. Materials and Methods

### 2.1. MFSR Signal Processing

High-frequency ultrasound transducers have inherent difficulties detecting blood vessels. Blood backscatter levels increase with frequency, causing distortion of the arterial lumen and increasing the difficulty of discerning blood vessels during biopsy procedures [82]. Although blood backscatter rises sharply as the transducer center frequency increases, the backscatter from vessel wall tissues grows more gradually [82,83]. These distinct blood and tissue properties were leveraged to enhance contrast between the arterial lumen and surrounding tissues, achieved by computing the ratio of low-frequency–filtered ultrasound signals to high-frequency signals [70,84,85].

In other words, MFSR is a contrast metric that compares ultrasound echo amplitudes at two different frequency ranges measured at the same spatial location. This comparison enhances blood–tissue discrimination.

Raw RF A-lines were extracted from each rotational (*α*) and axial (z) position and pre-processed in the following manner: first, the initial portion of each A-line was trimmed to suppress ringdown effects. Next, the respective means were subtracted from each signal to remove any DC bias. Following the pre-processing steps, two signal processing pipelines were utilized: MFSR and B-mode.

For the MFSR computations, blood–tissue contrast at different frequencies was emphasized using a two-band envelope ratio. Each A-line was bandpass filtered into a low band (20–25 MHz) and a high band (50–100 MHz) using the MATLAB (MATLAB R2024b, The MathWorks, Natick, MA, USA) function. Afterwards, the raw ratio was computed at each time and spatial position as:

(1) $$MFSR_{raw}\left(r,\alpha ,z\right)=\;\frac{E_{low}(r,\alpha ,z)}{max(E_{high}\left(r,\alpha ,z\right),\varepsilon )},$$

with $\varepsilon =10^{-12}$ to avoid division by zero. $E_{low}$ is the enveloped low-band (20–25 MHz) filtered signal, selected based on the measured pulse-echo bandwidth of the transducer and to suppress high-frequency blood-scattering effects while maintaining spectral separation with the high-band filtered signal. $E_{high}$ is the enveloped high-band (50–100 MHz) filtered signal, chosen to emphasize frequency-dependent attenuation and scattering differences between blood and surrounding tissue. After the raw MFSR ratio was calculated, it was normalized by the global volumetric maximum and converted to decibels.

### 2.2. Transducer Design and Fabrication

**Figure 1:**
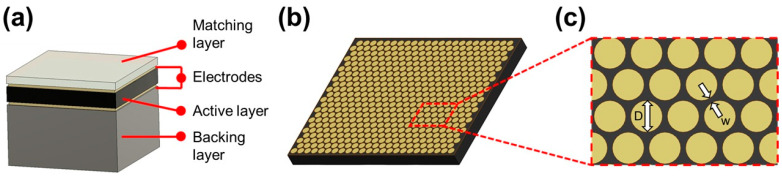
(**a**) Ultrasound transducer structure. (**b**) The 1–3 composite structure consisting of piezoelectric pillars embedded in an epoxy matrix. (**c**) Zoomed-in schematic of 1–3 composite structure, demonstrating pillar diameter, D, and kerf width, w.

For this study, PMN-PT 1–3 composite was selected as the active layer during the transducer design process due to its exceptional electromechanical coupling factor and low acoustic impedance, which leads to enhanced sensitivity and impedance matching with biological tissues, respectively. The epoxy matrix surrounding the embedded piezoelectric pillars reduces lateral and longitudinal excitation modes, increasing the bandwidth [86]. Broad bandwidth is especially important for MFSR imaging due to its use of low-band (20–25 MHz) and high-band (50–100 MHz) bandpass filters. The structure of 1–3 composite materials is shown in Figure 1b, with Figure 1c showing a zoomed-in view to reveal essential 1–3 composite parameters such as pillar diameter, D, and kerf width, w. The 1–3 composite was designed using the effective medium model (EMM), which simplifies the physics of the material under the assumption that it may be regarded as a homogeneous medium with adjusted material parameters such as density, elasticity, and dielectric permittivity [87]. Once the composite material properties were estimated, the transducer was designed with the Krimholtz, Leedom, and Matthaei (KLM) model to determine thickness, aperture size and materials used for the matching and backing layers [88]. A diagram of the ultrasound transducer structure is shown in Figure 1a. Silver epoxy (E-solder 3022, Von-Roll Inc., Cleveland, OH, USA) was selected for the backing layer due to its relatively high attenuation ratio and parylene c was chosen as the matching layer. Design details for each layer of the transducer are displayed in Table 1 and additional details regarding the high-frequency 1–3 composite transducer design and fabrication may be found in our previous publication [67].

**Figure 2:**
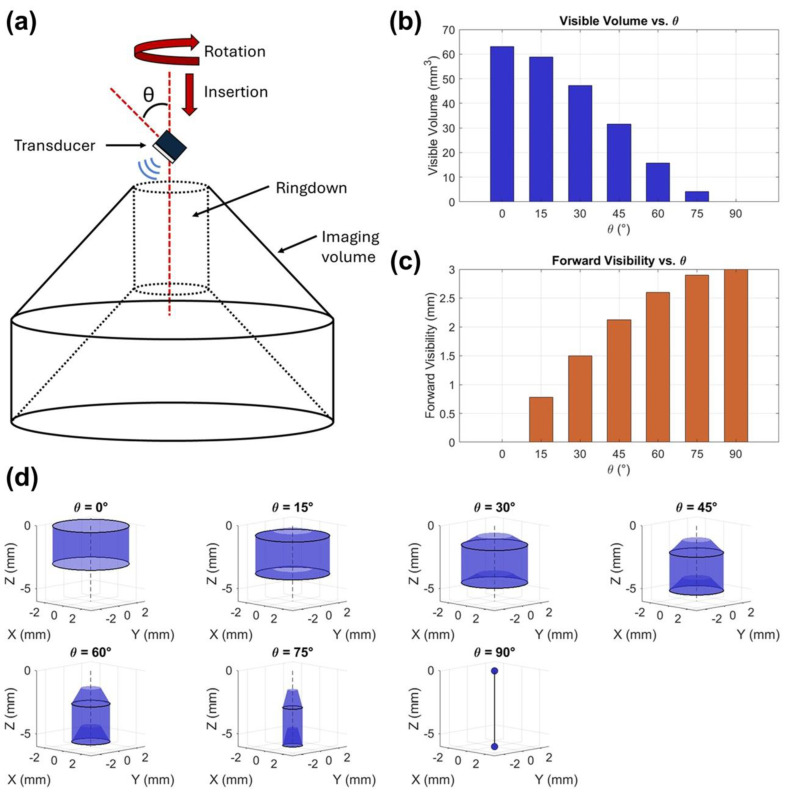
Effect of transducer angle on forward-viewing and volumetric imaging capabilities. (**a**) Schematic showing orientation of transducer angle (θ) with respect to insertion (along dotted line) and rotation directions. (**b**) Visible volumes calculated for various transducer mounting angles (0° ≤ θ ≤ 90°). Linear insertion depth and time of flight are 3 mm and 4 µs, respectively. (**c**) Forward visibility calculated for various transducer mounting angles (0° ≤ θ ≤ 90°). (**d**) Visualization of volumes constructed for various transducer mounting angles (0° ≤ θ ≤ 90°). Ringdown of 2 µs is accounted for.

To determine an optimal transducer mounting angle that balances forward-looking visibility and volumetric imaging coverage, a MATLAB script was developed to calculate the visible imaging volume and forward-viewing distance as a function of the transducer tilt angle, θ, relative to the needle shaft. Figure 2a shows the transducer tilt angle relative to the rotation and insertion directions of the needle. The code defined the imaging geometry as a truncated conical volume constrained by the total length of acquired A-lines (approximately 3 mm) and adjusted for a 2 µs ringdown to remove noise near the aperture of the device. For each tested angle (0–90° in 15° increments), the script computed effective visible volume by subtracting the ringdown region and quantified forward visibility as the axial projection of the acoustic beam along the insertion direction. Bar graphs shown in Figure 2b,c were generated to visualize the trade-off between total visible volume, which decreased with larger θ, and forward visibility, which increased with θ. Three-dimensional renderings in Figure 2d show the imaging volumes corresponding to this transition from side-looking to forward-oriented geometries. From this information, it was deduced that a 45° orientation was an effective compromise, providing over 30 mm^3^ of volumetric coverage while extending forward field of view to over 2 mm for improved vessel detection and needle navigation during biopsy procedures.

The selected 45° transducer orientation was implemented by integrating the single-element ultrasound transducer into an 18-gauge biopsy needle tip using a jig, which was 3D printed from polycarbonate to control the angle between the transducer backing and needle shaft during assembly. The transducer was bonded to the needle tip using non-conductive steel-reinforced epoxy (J-B Weld, Marietta, GA, USA). The transducer element was positioned flush with the outer bevel surface while maintaining clearance within the needle bore, ensuring the tissue collection pathway remained unobstructed. A 24-gauge coaxial cable was routed through the lumen of the needle shaft to provide electrical connection between the transducer electrodes and the external driving and receiving electronics.

### 2.3. Transducer Characterization

**Figure 3:**
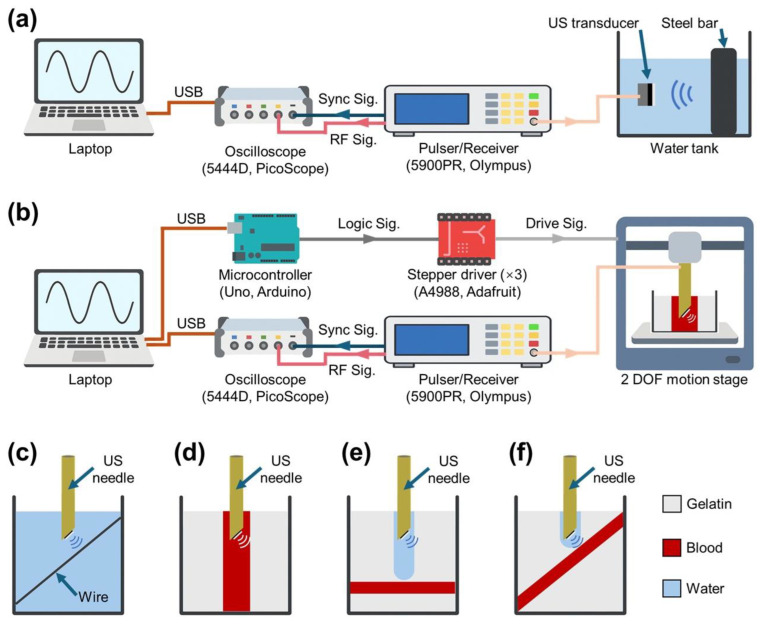
Illustration of the ROSUS experimental workflows. (**a**) Pulse-echo test experiment setup diagram. (**b**) Volumetric scanning experiment setup diagram. (**c**–**f**) Phantom schematics for the wire phantom and vessel phantoms (parallel, perpendicular and oblique orientations of needle with respect to vessel), respectively.

The electrical impedance spectrum and phase response of the high-frequency PMN-PT 1–3 composite transducer were characterized using an impedance analyzer (4294A, Agilent Technologies, Santa Clara, CA, USA) over the frequency range of 10–100 MHz. Pulse-echo testing was conducted in a degassed water tank to measure the transducer’s sensitivity and bandwidth. The device was excited by a pulser/receiver (5900PR, Olympus, Center Valley, PA, USA), and the reflected signals were recorded on a high-speed oscilloscope (PicoScope 5444D, Pico Technology, St Neots, Cambridgeshire, UK). The Picoscope was operated at a sampling rate of 200 MS/s with 8-bit vertical resolution. During testing, a polished stainless-steel reflector was positioned 0.7 mm from the transducer aperture. The experimental setup for pulse echo testing is shown in Figure 3a. Averaged echo waveforms were acquired on a laptop and analyzed offline to determine the center frequency, −6 dB bandwidth, and loop sensitivity [67].

The spatial resolution of the needle-integrated transducer was evaluated using a 160 µm diameter copper wire mounted at a 45° angle relative to the degassed water tank, so that the cross-section of the wire was positioned parallel to the transducer aperture. This orientation minimized geometric misalignment and provided a well-defined point target for imaging. The wire and water tank orientation are shown in Figure 3c. Mechanical scanning was conducted using a two-degree-of-freedom (DOF) motion stage that controlled both linear insertion and rotational motion of the needle. The insertion increment was 0.08 mm, and the rotation increment was 3.6° per step, corresponding to 100 A-lines per revolution. Data acquisition on the oscilloscope was synchronized with the stepper-motor controller so that each motion step corresponded to precisely one average A-line signal. A schematic for the experiment setup is depicted in Figure 3b. The acquired echo data were processed offline in MATLAB. These axial and lateral resolution measurements represent transducer characterization obtained with standard device evaluation practices.

### 2.4. Phantom Design and Fabrication

Tissue-mimicking phantoms with embedded blood vessel lumens were fabricated to model the acoustic interface between blood and surrounding soft tissues. Degassed water was first heated to 49 °C and continuously stirred while 5% m/V gelatin (G2500, Sigma-Aldrich, St. Louis, MO, USA) was gradually added. Once fully dissolved and transparent, the temperature was reduced to 38 °C and 1% m/V 20 µm cellulose particles (S3504, Sigma-Aldrich Corp., St. Louis, MO, USA) were introduced to provide controlled ultrasound scattering and attenuation comparable to that of biological tissue. The 1% concentration was selected based on prior phantom studies showing that cellulose loadings near 0.6–1% m/V yield realistic backscatter without excessively stiffening the matrix [89,90]. After mixing and degassing, the solution was injected into a mold containing a removable 4 mm diameter tube positioned to define a vessel lumen within the phantom. The phantom was refrigerated at 4 °C for 24 h to allow crosslinking, after which the rod was withdrawn, and the lumen was filled with fresh bovine blood to replicate the acoustic impedance and attenuation contrast between vascular and extravascular regions.

To evaluate ROSUS performance under different clinical insertion conditions, three phantom geometries were prepared. Figure 3d–f present the various phantom geometries, with Figure 3d showing a parallel angle between the needle shaft (insertion direction) and the vessel channel, Figure 3e showing a perpendicular angle between the needle shaft and the vessel wall, and Figure 3f showing an oblique angle configuration between the needle shaft and vessel wall. These vessel orientations enabled assessment of both side- and forward-looking imaging capabilities and contrast sensitivity across varying incidence angles at the blood–tissue interface.

### 2.5. Ultrasound Data Acquisition and Motion Control

Ultrasound data for the ROSUS phantom experiments were acquired using a synchronized MATLAB–Arduino–Picoscope workflow that coupled motion control and signal acquisition into a unified 3D scanning routine. The PicoScope 5444D was used for data acquisition in block-capture mode, while an Arduino (Arduino Uno, Arduino, Ivrea, Italy) microcontroller controlled the rotational and axial motion of the needle assembly through three A4988 (A4988 Stepper Motor Driver, Adafruit Industries, New York, NY, USA) stepper-motor drivers (one for rotation and two mechanically coupled for axial translation). The pulser/receiver excited the needle-integrated transducer, and its synchronization output was connected to the external trigger input of the PicoScope to ensure the deterministic timing between ultrasonic excitation and data capture. This experimental setup is depicted in Figure 3b.

The scanning protocol was fully automated through serial communication between MATLAB and the Arduino. Before initiating a scan, the operator could specify the first rotation direction and perform coarse (10 mm) or fine (1 mm) axial positioning through serial commands. Once the PicoScope was configured, the Arduino script executed rotational and axial motion in a serpentine pattern to minimize cable twisting, consisting of 100 angular increments per revolution (3.6° per increment, achieved through 4 microsteps per increment) and 60 axial layers spaced by 80 µm, corresponding to a total axial range of 4.8 mm. After each rotational increment, the Arduino paused to allow for mechanical stabilization and sent confirmation back to MATLAB over the serial interface. MATLAB detected the serial trigger and initiated an acquisition sequence, ensuring temporal alignment between each A-line and its corresponding position. Each of these acquisition sequences resulted in a 4 µs acquisition window on Channel A of the PicoScope, configured for DC coupling and a 2 V range at 8-bit resolution. Channels B–D were disabled to allow full bandwidth of the oscilloscope to be utilized. The PicoScope was triggered on the falling edge of the external synchronization pulse with a threshold of 3 V. To improve the signal-to-noise ratio, 12 consecutive A-lines were recorded and averaged before storage. The resulting data were stored in a pre-allocated three-dimensional array of size 60 × 100 × N, where N represents the number of time samples per A-line (4000). Each dataset was saved as a matrix file containing the volumetric RF data and acquisition metadata, including sampling interval and initial rotation direction. Each phantom imaging case was performed as a controlled proof-of-concept experiment to evaluate blood–tissue contrast mechanisms.

### 2.6. Volume Reconstruction Algorithm

A MATLAB-based volumetric reconstruction was implemented that converts the acquired ultrasound data into a truncated conical volume for visualization. For each slice in the z-direction, ringdown time is discarded by trimming a predetermined number of samples at the beginning of the A-lines, close to the transducer aperture. The remaining time-of-flight data is converted to depth in millimeters using the assumed speed of sound (1520 m/s). Depth samples r (mm) are then mapped to a conical coordinate system reflecting the fixed transducer tilt angle, θ. The radial reach of the volume in the transverse plane is ρ=rsinθ and the axial coordinate along the needle insertion direction is $z=-(r\cos\theta +z_{0})$, where $z_{0}$ advances by the known axial step between slices (80 µm). For each rotation angle α in [0, 2π), Cartesian coordinates are assembled as

(2) x=ρcosα, y=ρsinα, z (as above),

yielding a frustum shell per slice in the axial direction with top and bottom surfaces separated by the axial step size. These surfaces and their side walls are triangulated by reusing the slice’s intensity image. Since the motor alternates between clockwise and counterclockwise directions during the mechanical scanning, every other slice is flipped along the angle dimension prior to mapping to mitigate seam artifacts and allow direct stacking of each frustum shell.

Volumes are visually rendered using a graphical user interface (GUI) which allows the user to rotate the volume about *x*-, *y*-, or *z*-axes in a 3D Cartesian grayscale intensity plot for interactive visibility. Rendering is performed with semi-transparent frustum surfaces (surface opacity of 10%) to reveal internal structure without full volume ray-casting. For each slice in the z-direction, the script draws the top and bottom frustum surfaces, as well as the side walls formed from their connection in the depth direction. All surfaces share face colors obtained from the dynamic range values in decibels. Since the volumes are plotted with large 60 × 100 × 4000 arrays, they contain many voxels and are graphically demanding on the computer hardware. Therefore, a dedicated graphics card (NVIDIA GeForce RTX 4060 8 GB GPU, NVIDIA Corp., Santa Clara, CA, USA) is used while running the script.

### 2.7. B-Mode Signal Processing

The B-mode signal processing included normalizing each RF A-line by the maximum global reference, followed by conversion to decibels. For fair comparison, both MFSR and B-mode volumetric images used a dynamic range of 25 dB.

To quantitatively compare the contrast between MFSR and B-mode volumetric images, the contrast-to-noise ratio (CNR) was computed from the datasets using two regions of interest (ROIs). A MATLAB script was written to build a GUI for ROI selection and consequent CNR calculation from volumetric images. In the GUI menu, the user selects an image, followed by rectangular ROI selections. The first ROI selected is the tissue (gelatin/cellulose) region. The second ROI is the blood region. Next, the CNR is calculated as:

(3) $$CNR=\frac{\left|\mu_{tissue}-\mu_{blood}\right|}{\sqrt{\sigma_{tissue}^{2}+\sigma_{blood}^{2}}},$$

with μ being the average of the pixel intensities inside the selected ROI and σ being the standard deviation of the pixel intensities inside the selected ROI [91]. To avoid bias, the same ROIs (location and size) were used for B-mode and MFSR volumes acquired from the same phantoms. CNR was then reported for three ROIs in each respective imaging case.

### 2.8. Proposed Clinical Workflow

ROSUS is intended to provide local vessel proximity information during percutaneous biopsy. During the procedure, the operator advances the needle using standard clinical technique while acquiring rotational sweeps. The processed output provides an MFSR indicator that can be displayed as 2D slices or 3D localized maps. If a vessel region is detected adjacent to or ahead of the needle path, the operator can pause to redirect the trajectory before sampling.

## 3. Results

### 3.1. Transducer Characterization

**Figure 4:**
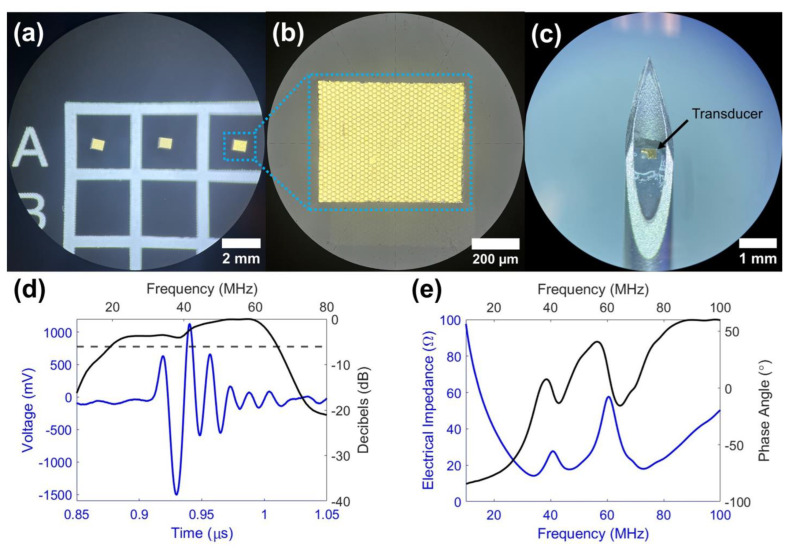
Transducer characterization results. (**a**) Top-view photograph of 1–3 composite transducers. (**b**) Zoomed-in top-view photograph of 1–3 composite transducer acoustic aperture. (**c**) Photograph of transducer and 18-gauge needle assembly. (**d**) Pulse-echo response of the transducer. (**e**) Electrical impedance spectrum of the transducer.

Figure 4 shows top-view photographs of the transducer aperture, a photograph of the transducer–needle assembly, the measured pulse-echo response, and the electrical impedance spectrum for the high-frequency single element ultrasound transducer. The electrical impedance spectrum and phase angle calibrated with cables are displayed in Figure 4e. The electrical impedance magnitude at resonance is 17.8 Ω, and the measured capacitance and dielectric loss are 189 pF and 44.2 mU, correspondingly. The pulse-echo response (Figure 4d) shows a received echo with a center frequency of 57.6 MHz and a −6 dB fractional bandwidth of 81.1%. Under a 2 μJ excitation, the echo amplitude is 2.63 V_pp_, corresponding to a loop sensitivity of −33.2 dB.

### 3.2. Resolution Measurement

**Figure 5:**
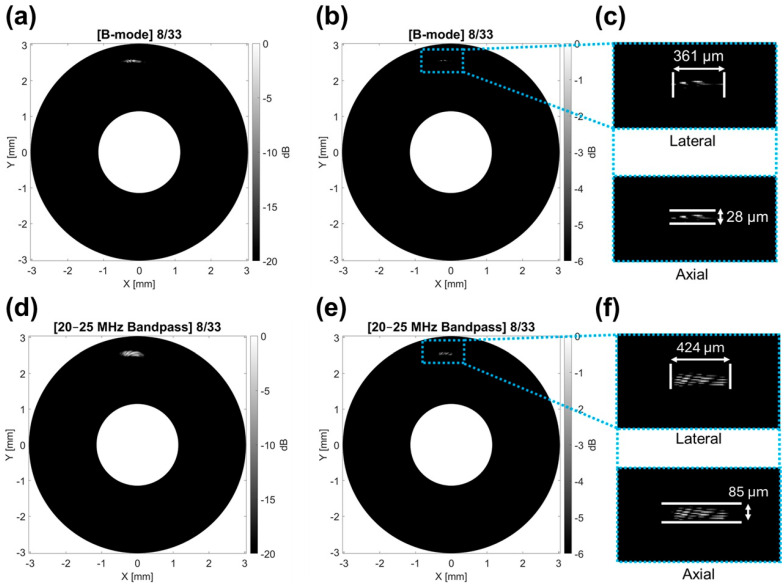
Transducer resolution measurement. (**a**) B-mode image plotted with 20 dB dynamic range. (**b**) B-mode image plotted with 6 dB dynamic range. (**c**) Measured lateral and axial resolutions from 6 dB B-mode image. (**d**) A 20–25 MHz bandpass filtered B-mode image plotted with 20 dB dynamic range. (**e**) A 20–25 MHz bandpass filtered B-mode image plotted with 6 dB dynamic range. (**f**) Measured lateral and axial resolutions from 6 dB 20–25 MHz bandpass filtered B-mode image.

Scans used to measure the axial and lateral resolutions of the transducer are depicted as polar B-mode images and shown in Figure 5. The resolution for standard B-mode imaging with full bandwidth is described by Figure 5a–c, with Figure 5a showing the wire position using a 20 dB dynamic range, Figure 5b showing the −6 dB intensity profile within the polar image, and Figure 5c showing the zoomed-in photo of the −6 dB intensity contour with corresponding measurements in the axial and lateral directions. A dynamic range of 20 dB has been shown to provide adequate contrast for phantom wire visualization and location, enabling reliable placement of the ROI for subsequent resolution measurement [92]. The −6 dB contours are then used for resolution measurement, as they correspond to the full width at half maximum (FWHM) of the point-spread function and represent the standard criterion for defining axial and lateral spatial resolution in ultrasound imaging [93,94]. The axial resolution was 28 µm and the lateral resolution was 361 µm. MFSR resolution is limited by the lower-frequency bandpass filter (20–25 MHz) due to the acoustic wavelength and focal width in that frequency regime being larger than those in the 50–100 MHz range. Therefore, to measure the MFSR resolution, the same dataset was used but filtered from 20–25 MHz to incorporate the limitation. With this lower-frequency bandpass filtered data, the MFSR resolution was measured to be 85 µm in the axial direction and 424 µm in the lateral direction.

### 3.3. Volume Reconstruction and MFSR Performance

**Figure 6:**
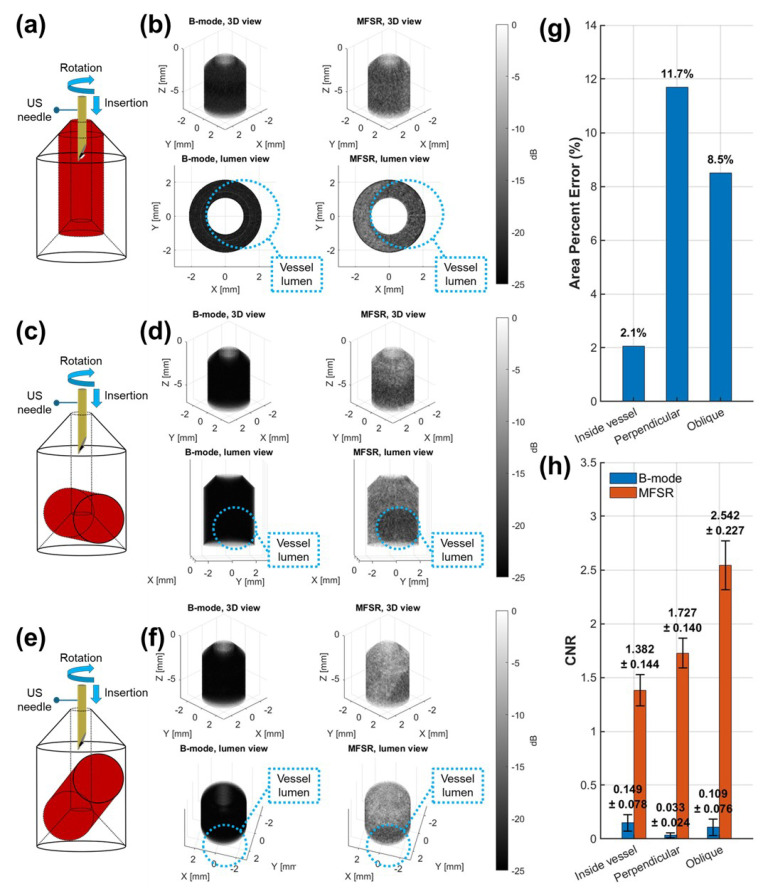
Volumetric images for B-mode versus MFSR signal processing algorithms. (**a**) 3D schematic depicting the needle inside the vessel. (**b**) Images with needle inside the vessel, including 3D view for B-mode (top left), 3D view for MFSR (top right), lumen view for B-mode (bottom left), and lumen view for MFSR (bottom right). (**c**) 3D schematic depicting the needle shaft perpendicular to the vessel. (**d**) Images with needle shaft perpendicular to the vessel, including 3D view for B-mode (top left), 3D view for MFSR (top right), lumen view for B-mode (bottom left), and lumen view for MFSR (bottom right). (**e**) 3D schematic depicting the needle shaft at an oblique angle with respect to the vessel. (**f**) Images with needle shaft at an oblique angle with respect to the vessel, including 3D view for B-mode (top left), 3D view for MFSR (top right), lumen view for B-mode (bottom left), and lumen view for MFSR (bottom right). (**g**) Measured area percent error for the vessel lumen with MFSR signal processing. (**h**) Measured CNR for B-mode versus MFSR signal processing algorithms. Bars represent mean ± standard deviation across three independent ROIs; CNR differences between B-mode and MFSR are statistically significant (Welch’s two-sample t-test, *p* < 0.01).

Volumetric images are shown alongside their respective 3D schematics for the scanning of each phantom in Figure 6a–f. For each phantom and corresponding needle–vessel orientation, B-mode and MFSR volumes are visualized with both standard 3D views and views of the vessel lumen cross-sections. Vessels were located using the MFSR vessel cross-section images and the vessel areas were compared with the cross-sectional areas of the cylinders used for molding the phantoms. The area percentage error was computed and shown in Figure 6g. The error was reasonable in all scenarios, with a maximum of 11.7% error measured when the needle was perpendicular to the vessel. Compared with B-mode volumetric images, the MFSR volumes showed drastically increased contrast. As shown in Figure 6h, the CNR for B-mode volumes never passed 0.25, yielding an indistinguishable vessel lumen from surrounding tissues. On the other hand, MFSR showed enough contrast to discern the vessel lumen and surrounding tissues, with measured CNR averages of 1.382, 1.727, and 2.542 for inside the vessel, perpendicular to the vessel wall, and an oblique angle with respect to the vessel wall, respectively. MFSR showed a significantly higher CNR than B-mode imaging for all three configurations (Welch’s two-sample t-test, *p* < 0.01). The increased CNR in the oblique angle case is likely observed due to the favorable angle of orientation between the transducer aperture and the vessel wall, which are near-parallel compared with the other two cases.

## 4. Discussion

This study presents ROSUS, which combines an obliquely mounted single-element ultrasound transducer with synchronized rotational–axial scanning and MFSR processing to enhance blood–tissue contrast during biopsy procedures. Volumetric renderings achieved higher vessel conspicuity than B-mode imaging, with CNR increasing from ≤ 0.25 (B-mode) to 1.38–2.54 (MFSR) across three representative needle–vessel geometries. Vessel lumen area errors were ≤11.7%, demonstrating quantitative accuracy sufficient for enhancing trajectory planning. Resolution testing confirmed 28 µm axial and 361 µm lateral resolution under full-band B-mode operation and 85 µm axial, 424 µm lateral resolution for the lower-frequency (20–25 MHz) band that forms the limitation of the MFSR.

A high-frequency transducer embedded directly within the biopsy needle tip overcomes the attenuation and motion dependencies that limit conventional external ultrasound and Doppler guidance. The 45° oblique mounting provides both forward- and side-looking perspectives, enabling visualization of vessels along and ahead of the needle trajectory. Unlike US array- or optics-based modalities, ROSUS leverages a single-element transducer for assembly simplicity and volumetric mapping through serpentine scanning. This configuration, combined with frequency-domain backscatter contrast rather than flow detection, delivers vessel delineation without requiring multielement arrays. Because ROSUS does not rely on flow-induced frequency shifts it is not inherently dependent on blood velocity or flow direction. Instead, the MFSR contrast mechanism depends on frequency-dependent backscatter and attenuation properties, with blood flow expected to primarily introduce increased speckle variability rather than systematic loss of contrast [95]. While Doppler and CEUS can enhance vascular visualization, they rely on array transducers or contrast agents that are incompatible with miniaturized, needle-based systems. Therefore, ROSUS serves as a complementary approach optimized for constrained interventional geometries, with trade-offs in temporal resolution and quantitative flow measurement. A comparison of these modalities is provided in Table 2. Furthermore, side-looking OCT needles are limited by optical scattering to shallow penetration depth on the order of 1–3 mm in tissue and lack forward-viewing capability, restricting visualization of vessels along the needle trajectory [64,96,97]. Photoacoustic approaches offer strong hemoglobin-based contrast, but their imaging range is constrained by optical attenuation and fluence limits [62]. They also require integration of both optical and acoustic modalities, increasing system complexity compared to ultrasound-only modalities [62,63].

The differences in CNR among the three phantom geometries primarily stem from variations in insonation angle and acoustic propagation path. In the oblique configuration, the transducer aperture was nearly parallel to the vessel wall, producing strong specular reflections and enhanced spectral separation between blood and tissue. Therefore, using MFSR signal processing, the oblique geometry accentuates the contrast between the two mediums, producing the highest CNR average (2.54). In the perpendicular orientation, the insonation angle reduces coherent reflection from the vessel wall, leading to diminished low-frequency dominance and a moderate CNR average (1.73). The inside-vessel configuration produced the lowest CNR average (1.38) since the ultrasound propagated through blood before encountering the wall, allowing the high-frequency band to dominate earlier and thus compressing the MFSR. These behaviors are consistent with the angular dependence of backscatter at soft-tissue interfaces and the differential attenuation of tissue and blood across frequency bands [82,98,99,100].

The lumen area estimation errors are attributed largely to geometric factors in cross-section extraction rather than intrinsic imaging limitations. In the 3D volume reconstruction GUI, the inside-vessel case corresponded to the top view of the volume (90° elevation), where the lumen cross-section was directly aligned with the default viewing plane, minimizing interpolation or projection errors. Conversely, the perpendicular and oblique cases required rotating the volumetric view to locate the lumen cross-section, introducing small misalignments between the true vessel axis and the extracted slice. These orientation-related discrepancies slightly distorted the measured lumen boundary and, thus, inflated area error. Additionally, the longer effective propagation paths through tissue in these two cases likely increased high-frequency attenuation and reduced wall definition, compounding segmentation uncertainty [100,101]. These effects explain the rise in area error from the inside-vessel to oblique geometries while remaining in a reasonable range, confirming the geometric fidelity of the volumetric reconstruction.

At ultrasonic frequencies above 20 MHz, the backscattering coefficient of blood increases rapidly with frequency, approximately following an $f^{4}$ dependence in the Rayleigh regime [82,83]. Soft-tissue interfaces, in contrast, show a more gradual backscatter shift with respect to frequency in this range [82]. ROSUS capitalizes on this disparity by band splitting each A-line into low (20–25 MHz) and high (50–100 MHz) components and forming their envelope ratio [70,84,85]. Regions with a relatively stronger low-frequency response, such as tissue interfaces, appear brighter, whereas blood, which scatters more at higher frequencies, yields lower ratio values. The experimentally observed CNR increase agrees with this hypothesis and confirms that spectral contrast can substitute for Doppler or microbubble contrast agents where flow or perfusion is absent.

The darker appearance and limited speckle detail in the reconstructed B-mode volumes primarily arise from frequency-dependent attenuation, which reduces backscatter amplitude from deeper tissue and blood regions. Additional visualization factors also contribute: both B-mode and MFSR volumes were rendered with 10% surface opacity to enable viewing of internal features within the 3D reconstructions. Furthermore, each dataset was normalized to its global maximum intensity, causing a few stronger echoes to dominate the dynamic range and suppress lower-amplitude speckle.

ROSUS directly addresses a major complication of percutaneous biopsy procedures—inadvertent vessel puncture leading to hemorrhage [15,16,17,18,19,20]. By providing 3D volumetric visualization and enhanced vessel contrast without Doppler angle/array constraints or intravenous agents, the technique can inform adjustments to the needle trajectory and potentially reduce the number of passes required for diagnostic sampling. There are still limitations to this work. A full 3D scan requires approximately 50 min, with the mechanical scanning rate deliberately limited by synchronization and motor settling time. Faster acquisition could be achieved through continuous rotation of the needle during data acquisition. Prior work in percutaneous needle interventions has demonstrated that controlled needle rotation at 50–100 revolutions per minute (RPM) is feasible and compatible with standard biopsy workflows [102]. In the present implementation, including 12 averages per spatial location, a rotation speed of 60 RPM would allow a full ROSUS volume reconstruction in 12 min. This acquisition timescale is well aligned with typical ultrasound-guided percutaneous biopsy procedures, which commonly require approximately 5–15 min for needle placement and sampling [103,104,105]. The current study was evaluated exclusively using static gelatin–cellulose–blood phantoms and has not yet been tested in patients or in vivo. These phantoms replicate the acoustic scattering of blood and tissue interfaces. High-frequency ultrasound attenuates quickly in heterogeneous tissue, but this effect is mitigated in ROSUS by positioning the transducer at the needle tip, minimizing propagation distance. Furthermore, since the MFSR is a ratio of low- and high-frequency backscatter at the same spatial location, it reduces sensitivity to attenuation in soft tissues, emphasizing relative spectral behavior rather than absolute signal magnitude. Yet an upgraded perfused phantom setup or in vivo testing is needed to assess performance under pulsatile and heterogeneous conditions. The obliquely mounted transducer is fixed into the needle tip using steel-reinforced epoxy, and future iterations will evaluate the structural stability under insertion loads and cyclic use. Notably, the needle-integrated transducer assembly remained intact throughout hundreds of insertion and rotation steps inside the phantoms during the experiments performed, with no observed degradation to the structure or acoustic performance. Electrical interference in clinical environments is mitigated using coaxial cabling, consistent with standard high-frequency ultrasound system design. Machine learning techniques and further quantitative ultrasound (QUS) classifiers may be introduced to further enhance the CNR.

## 5. Conclusions

A rotational oblique spectral ultrasound (ROSUS) system integrating a 57.5 MHz PMN-PT 1–3 composite transducer mounted at a 45° angle within the needle tip achieved 85 µm axial and 424 µm lateral resolutions. The oblique transducer configuration enabled simultaneous forward- and side-viewing perspectives along the needle trajectory. MFSR processing increased vessel–tissue CNR from ≤0.25 (B-mode) to 1.38–2.54. The compact needle-integrated design supports volumetric vascular visualization for image-guided biopsy. Future work will focus on real-time reconstruction and advanced phantom validation.

## Figures and Tables

**Table 1:** Design parameters for single-element 1–3 composite transducer.

	Material	Thickness	Velocity	Density	Acoustic Impedance
Active layer	PMN-PT 1–3 composite	21 μm	3890 m/s	5272 kg/m^3^	20.5 MRayl
Matching layer	Parylene C	8 μm	2770 m/s	1140 kg/m^3^	3.16 MRayl
Backing layer	E-solder 3022	500 μm	2110 m/s	2590 kg/m^3^	5.5 MRayl

**Table 2:** Comparison of ROSUS with Doppler and CEUS [49,50,51,52,53,54,55,56].

Feature	Doppler US	CEUS	ROSUS
Mechanism	Flow-induced frequency shift	Microbubble perfusion	Frequency-dependent backscatter
Flow dependence	Yes	Indirect (perfusion)	No
Contrast agents	No	Yes	No
Hardware requirement	Array	Array	Single-element
Primary output	Flow velocity	Vascular enhancement	Blood–tissue contrast
Real-time capability	Yes	Yes	Feasible with continuous rotation
Resolution regime	Sub-mm- to mm-scale (array dependent)	Sub-mm- to mm-scale (array-dependent)	Tens of µm axial; sub-mm lateral
Penetration depth regime	cm-scale	cm-scale	mm-scale to cm-scale

## Data Availability

The data presented in this study will be made publicly available and/or is available upon request from the corresponding authors.
